# Double inversion recovery MRI versus contrast-enhanced MRI for evaluation of knee synovitis in juvenile idiopathic arthritis

**DOI:** 10.1186/s13244-022-01299-0

**Published:** 2022-10-20

**Authors:** Floris Verkuil, Robert Hemke, E. Charlotte van Gulik, Anouk M. Barendregt, Amara Nassar-Sheikh Rashid, Dieneke Schonenberg-Meinema, Koert M. Dolman, Eline E. Deurloo, Kees F. van Dijke, J. Michiel den Harder, Taco W. Kuijpers, J. Merlijn van den Berg, Mario Maas

**Affiliations:** 1 Radiology and Nuclear Medicine, Amsterdam Movement Sciences, Amsterdam UMC Location University of Amsterdam, Meibergdreef 9, 1105 AZ Amsterdam, The Netherlands; 2 Pediatric Immunology, Rheumatology and Infectious Diseases, Emma Children’s Hospital, Amsterdam UMC Location University of Amsterdam, Meibergdreef 9, 1105 AZ Amsterdam, The Netherlands; 3 Department of Pediatrics, Zaans Medical Center, Koningin Julianaplein 58, 1502 DV Zaandam, The Netherlands; 4 Department of Pediatrics; Location OLVG Oost, OLVG, Oosterpark 9, 1091 AC Amsterdam, The Netherlands; 5 Department of Pediatrics; Location OLVG West, OLVG, Jan Tooropstraat 164, 1061 AE Amsterdam, The Netherlands; 6 Pediatric Rheumatology, Reade, Dr. Jan van Breemenstraat 2, 1056 AB Amsterdam, The Netherlands; 7 Department of Radiology and Nuclear Medicine, Noordwest Hospital Group Alkmaar, Wilhelminalaan 12, 1815 JD Alkmaar, The Netherlands

**Keywords:** Juvenile idiopathic arthritis, Synovitis, Double inversion recovery MRI

## Abstract

**Background:**

Double inversion recovery (DIR) MRI has the potential to accentuate the synovium without using contrast agents, as it allows simultaneous signal suppression of fluid and fat. The purpose of this study was (1) to compare DIR MRI to conventional contrast-enhanced (CE) MRI for delineation of the synovium in the knee in children with juvenile idiopathic arthritis (JIA) and (2) to assess the agreement between DIR MRI and CE-MRI regarding maximal synovial thickness measurements.

**Results:**

In this prospective study, 26 children with JIA who consecutively underwent 3.0-T knee MRI between January 2018 and January 2021 were included (presence of knee arthritis: 13 [50%]; median age: 14 years [interquartile range [IQR]: 11–17]; 14 girls). Median confidence to depict the synovium (0–100 mm visual analogue scale; scored by 2 readers [consensus based]) was 88 (IQR: 79–97) for DIR MRI versus 100 (IQR: 100–100) for CE-MRI (*p* value = < .001). Maximal synovial thickness per child (millimeters; scored by 4 individual readers) on DIR MRI was greater (*p* value = < .001) in the children with knee arthritis (2.4 mm [IQR: 2.1–3.1]) than in those without knee arthritis (1.4 mm [IQR: 1.0–1.6]). Good inter-technique agreement for maximal synovial thickness per child was observed (*r*_s_ = 0.93 [*p* value = < .001]; inter-reader reliability: ICC DIR MRI = 0.87 [*p* value = < .001], ICC CE-MRI = 0.90 [*p* value = < .001]).

**Conclusion:**

DIR MRI adequately delineated the synovium in the knee of children with JIA and enabled synovial thickness measurement similar to that of CE-MRI. Our results demonstrate that DIR MRI should be considered as a child-friendly alternative to CE-MRI for evaluation of synovitis in children with (suspected) JIA.

**Supplementary Information:**

The online version contains supplementary material available at 10.1186/s13244-022-01299-0.

## Key points

- Double inversion recovery (DIR) MRI adequately delineated the synovial lining in the knee joint.
- The performance of DIR MRI to measure the maximal synovial thickness per child corresponded to that of conventional CE-MRI.
- DIR MRI demonstrated potential to discriminate present knee arthritis from absent knee arthritis.

## Background

Currently, MRI is advocated as the preferred imaging modality for diagnosis and monitoring of juvenile idiopathic arthritis (JIA) [1], as it enables simultaneous detailed visualization of all JIA-relevant structures [1–4]. The hallmark feature of JIA disease activity is synovial inflammation [5]. Identification of the synovial lining on standard unenhanced MRI sequences is challenging [6], since synovium and effusion generally have similar signal intensities on conventional T1- and T2-weighted images. Consequently, the sensitivity for detection of pathologic synovial thickening on non-enhanced MRI is limited [7]. To improve visibility of the synovium, use of contrast agents is recommended [7]. However, intravenous contrast administration is invasive, time-consuming, expensive [8, 9], and associated with potential side effects (e.g., allergic reaction, nephrogenic systemic fibrosis, and gadolinium deposition in the brain) [10]. To improve accessibility of MRI in JIA management, non-enhanced MRI methods able to accurately assess the synovium are crucial.

An MRI sequence that could meet these demands is double inversion recovery (DIR). This technique, primarily applied in neuroimaging [11–17], uses two 180° inversion pulses to selectively null the signals from two distinct tissue types with different T1 relaxation times [18, 19]. DIR MRI for delineation of the synovium has recently been explored in small cohorts of adults with various afflictions of the knee [20–22]. The findings of these pilot studies demonstrate the potential of DIR MRI to identify synovitis [20–22]. Nevertheless, the value of DIR MRI for assessment of the synovium in pediatric joints is unknown.

We hypothesized that DIR MRI adequately accentuates the synovium in the knee of children with JIA and enables adequate evaluation of synovial thickening to detect synovitis. Therefore, the purpose of this study was (1) to compare DIR MRI to conventional contrast-enhanced (CE) MRI for delineation of the synovium in the knee in children with JIA and (2) to assess the agreement between DIR MRI and CE-MRI regarding maximal synovial thickness measurements.

## Materials and methods

The current study is part of the Amsterdam Juvenile Arthritis Cohort Studies, a multicenter study designed to investigate the value of imaging markers as well as immunological markers for assessment of JIA disease activity. The three participating pediatric rheumatology centers were: Amsterdam University Medical Centers (location: Academic Medical Centre), Reade and Onze Lieve Vrouwe Gasthuis, all in Amsterdam, the Netherlands. Clinical data were obtained in the three centers. In order to optimize uniformity in imaging acquisition, MRI scanning was performed in one center (i.e., Amsterdam University Medical Centers [location: Academic Medical Centre]).

### Study participants

**Fig. 1:**
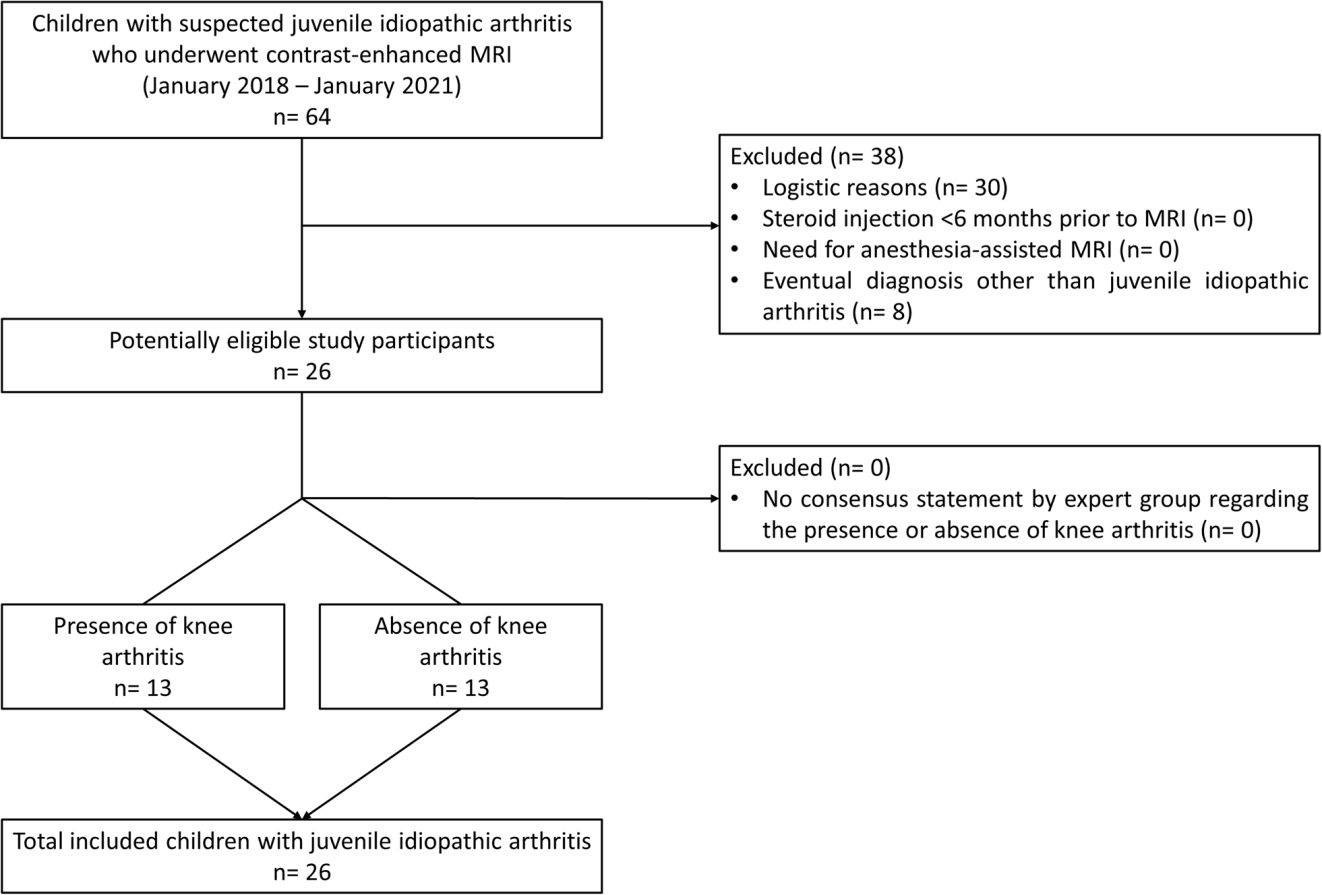
Study participant flow diagram

From January 2018 until January 2021, all consecutive children diagnosed with JIA who underwent knee MRI were prospectively included. A study participant flow diagram is shown in Fig. 1. The diagnosis of JIA was made by one of four pediatric rheumatologists (A.N., D.S., J.M.v.d.B., and K.M.D.: range of years’ experience = 5–17) according to the International League of Associations for Rheumatology (ILAR) criteria [23]. The decision to perform MRI examination was based on clinical indication. Exclusion criteria were: (1) intra-articular corticosteroid injection within the last six months and (2) need for anesthesia-assisted MRI. A waiver of informed consent was granted by the local ethics committee for this specific study.

### Clinical assessment

Prior to MRI examination, all children underwent clinical evaluation. Physical examination included a joint-specific assessment of tenderness, swelling, and joint mobility for 71 joints in total. Physician’s global assessment of overall disease activity was measured on a 100 mm visual analogue scale [24]. Laboratory examination included erythrocyte sedimentation rate (ESR) and C-reactive protein (CRP).

### Reference standard for presence of knee arthritis

Based on the consensus statement by a multidisciplinary expert group [25], the included children with JIA were subdivided into two subgroups: ‘presence of knee arthritis’ and ‘absence of knee arthritis.’ The panel consisted of pediatric rheumatologists, a pediatric rheumatology nurse, and radiologists. During bimonthly multidisciplinary team sessions, the expert group discussed the clinical data (i.e., age, sex, medical history, JIA subtype, disease course, treatment effect, physical examination findings, and laboratory results) and imaging results (i.e., ultrasound, conventional radiography, and/or pre- and post-contrast MRI) of each child and subsequently determined whether knee arthritis was present or not. DIR images were not available during the meetings. Participant categorization preceded study image analysis.

### MRI protocol

Images were obtained using a 3.0-T Ingenia MRI scanner (Philips Medical Systems) equipped with a dedicated knee coil. Participants were situated in a supine position with the knee placed centrally in the magnetic field. Standard knee MRI scanning protocol included three-dimensional fat-saturated T1- and T2-weighted scans before contrast administration and three-dimensional post-contrast fat-saturated T1-weighted scans. The post-contrast fat-saturated T1-weighted scans, obtained < 5 min after intravenous gadolinium injection (0.1 mg/kg body weight; gadobutrol; Schering AG), were used for image analysis (scanning parameters are presented in Table 1).

**Table 1:** Parameters of the MRI sequences used for image analysis

Sequence	GBCA	TR (ms)	TE (ms)	TE spacing (ms)	TSE factor	FOV (mm)	Acquisition matrix	Section thickness (mm)	Slice gap (mm)	Reconstruction voxel size	Compressed SENSE reduction factor
DIR	No	8343	25	8	11	AP 140 × RL 140 × FH 98.69	280 × 207	3	0.3	0.365 × 0.365	4
T1-weighted^a^	Yes	7.9	2.4	1.7	N/A	AP 150 × RL 142 × FH 130	200 × 191	2.5	0	0.469 × 0.469	N/A

DIR, double inversion recovery; GBCA, gadolinium-based contrast agent; TR, repetition time; TE, echo time; TSE, turbo spin echo; FOV, field of view; AP, anterior/posterior direction; RL, right/left direction; FH, foot/head direction; SENSE, sensitivity encoding; N/A, not applicable

^a^Fat suppression

For the purpose of this study, an axial DIR pulse sequence was added to the routine scanning protocol and applied prior to contrast administration. Standard DIR sequence settings, embedded in the MRI Scanner software, were adjusted in accordance with the protocol presented by Jahng et al. [20] and Son et al. [21] (Table 1). To acquire adequate delineation of the synovium by simultaneous suppression of fat tissue and synovial effusion, we used the inversion times reported by Son et al. [21] (i.e., first inversion time = 2830 ms; second inversion time = 254 ms). Total scan duration of the DIR sequence was 2 min and 14 s.

### Image analysis

To avoid any bias, the MRI dataset was anonymized and randomized. The following study parameters were assessed in the axial plane: (1) confidence in visual identification of the synovial lining, (2) overlap of the synovial distribution patterns, and (3) maximal synovial thickness. In accordance with the validated Juvenile Arthritis MRI Scoring (JAMRIS) system for the knee, parameters were evaluated at the following 6 anatomical locations: patellofemoral, suprapatellar recesses, infrapatellar fat pad, cruciate ligaments, medial posterior condyle, and lateral posterior condyle [26]. Study variables were evaluated by radiologists (R.H., a musculoskeletal radiologist with 11 years of experience; K.F.v.D., a musculoskeletal radiologist with 22 years of experience; E.E.D., a pediatric radiologist with 13 years of experience; and M.M., a musculoskeletal radiologist with 22 years of experience), from two institutions, blinded to clinical data. Study measurements were carried out consecutively during one scoring session, unless otherwise stated.

To assess whether the DIR pulse sequence delineates the synovium in the knee in children with JIA, we assessed reader’s confidence (readers: R.H. and M.M.; [endpoint: consensus score]) in visual identification of the synovium on DIR MRI and compared it to conventional CE-MRI. Confidence to depict the synovial lining was rated on a visual analogue scale from 0 (no confidence) to 100 (maximal confidence) [25, 27]. To reduce potential effects of reader preferences, this specific study parameter was assessed on separate occasions (i.e., session 1 = DIR MRI; session 2 = CE-MRI), with a 4-week interval in between and MRI dataset re-randomization before start of the second scoring session. Pairing DIR scans with post-contrast images was prohibited.

To assess whether the area covered by the synovium on DIR MRI corresponds with the synovial signature on CE-MRI, the synovial distribution patterns on both imaging techniques were compared (readers: R.H. and M.M.; [endpoint: consensus score]) using aligned DIR/post-contrast image pairs [21]. DIR images were always presented on the left side of the screen. The degree of correspondence was evaluated as the estimated percentage of overlap of the synovial distribution patterns (i.e., ≤ 25%; 26–50%; 51–75%; > 75%).

Measurement of maximal synovial thickness using CE-MRI is still the only validated MRI tool for assessment of synovitis in the knee in children with JIA [26]. To evaluate if similar results can be obtained using DIR MRI, maximal synovial thickness was evaluated on DIR MRI and compared to corresponding findings acquired using CE-MRI. This specific study parameter was evaluated by 4 individual readers (R.H., K.F.v.D., E.E.D., and M.M.). To test intra-reader reliability for evaluation of the maximal synovial thickness on DIR MRI, measurements were repeated by the 4 readers after 3 weeks, to minimize recall bias, in all study participants.

To assess whether the value of DIR-derived maximal synovial thickness per child for detection of arthritis corresponds with CE-MRI, we compared the findings between the children with knee arthritis and those without knee arthritis.

### Statistical analysis

Sample size selection (i.e., at least 12 participants per group of interest) was based on the data provided by a feasibility study using DIR MRI for evaluation of the synovium in a small cohort of adults [21], and recommendations for studies with a pilot nature [28]. Subgroup comparisons were performed by using the Student's *t* test, Mann–Whitney *U* test, Kruskal–Wallis test, Chi-square test, and Fisher's exact test. The Wilcoxon signed-rank test and Friedman test were carried out to evaluate differences between paired data. Spearman’s rank-order correlation coefficients [29] were computed to assess the relationship between DIR MRI and CE-MRI regarding maximal synovial thickness measurements. Intraclass correlation coefficient’s (ICC’s), based on single-measurement, absolute-agreement, two-way random effects model, were used to evaluate inter-reader reliability regarding measurement of the maximal synovial thickness. To evaluate intra-reader reliability, we used Bland–Altman plots and ICC’s [30], based on a single-measurement, absolute-agreement, two-way mixed effects model. Two-sided *p* values were used for all statistical assessments and a *p* value < 0.05 was considered statistically significant. Statistical analyses were performed using SPSS Statistics for Windows version 26.0 (IBM) and GraphPad Prism 8.0 (GraphPad Software).

## Results

### Study participant characteristics

We included 26 children with JIA (presence of knee arthritis: 13 [50%], 14 girls, with a median age of 14 years (interquartile range [IQR]: 11–17). An overview of participant demographics and clinical disease activity parameters is displayed in Table 2.

**Table 2:** Baseline characteristics of children in the study sample

Characteristic	All children with JIA	Presence of arthritis	Absence of arthritis	*p* *value*
No. of children	26	13	13	
Girls	14 (54%)	9 (69%)	5 (39%)	.24^a^
Age at MRI in years	14.3 (11.2–16.5)	14.0 (9.8–17.0)	14.4 (11.6–15.7)	.78^b^
*JIA ILAR subtype*				
Oligoarthritis; persistent	16 (62%)	9 (69%)	7 (54%)	.69^a^
Oligoarthritis; extended	2 (8%)	1 (8%)	1 (9%)	> .99^a^
Polyarthritis; RF negative	7 (27%)	3 (23%)	4 (31%)	> .99^a^
Enthesitis-related arthritis	1 (4%)	0 (0%)	1 (8%)	> .99^a^
No. of clinically inflamed joints	1.0 (1.0–2.0)	1.0 (1.0–1.5)	2.0 (1.0–3.0)	.16^b^
No. of joints with limited range of motion	1.0 (0.0–2.0)	1.0 (1.0–1.5)	1.0 (0.5– 2.0)	.40^b^
Physician's global assessment of overall disease activity^c^	13.5 (5.0–17.8)	15.0 (5.0–19.0)	10.0 (1.5–15.0)	.15^b^
CRP, mg/l	0.5 (0.3–1.7)	1.3 (0.4–2.5)	0.3 (0.2–0.5)	.08^b^
ESR, mm/h	2.0 (2.0–8.5)	8.0 (2.0–11.5)	2.0 (2.0–2.8)	.08^b^
*Drug treatment*				
No medication	14 (54%)	7 (54%)	7 (54%)	> .99^a^
NSAIDs	6 (23%)	3 (23%)	3 (23%)	> .99^a^
MTX or other cDMARD	4 (15%)	2 (15%)	2 (15%)	> .99^a^
bDMARD	2 (8%)	1 (8%)	1 (8%)	> .99^a^

Data are displayed as numbers (frequencies) or median (interquartile range), unless otherwise stated

JIA, juvenile idiopathic arthritis; ILAR, International League of Associations for Rheumatology; RF, rheumatoid factor; CRP, C-reactive protein; ESR, erythrocyte sedimentation rate; CE, contrast-enhanced; NSAIDS, nonsteroidal anti-inflammatory drug; MTX, methotrexate; cDMARD, conventional disease-modifying antirheumatic drug; bDMARD, biologic disease-modifying antirheumatic drug

^a^Fisher’s exact test

^b^Mann–Whitney *U* test

^c^Visual analogue scale: 0 mm = best, 100 mm = worst

**Fig. 2:**
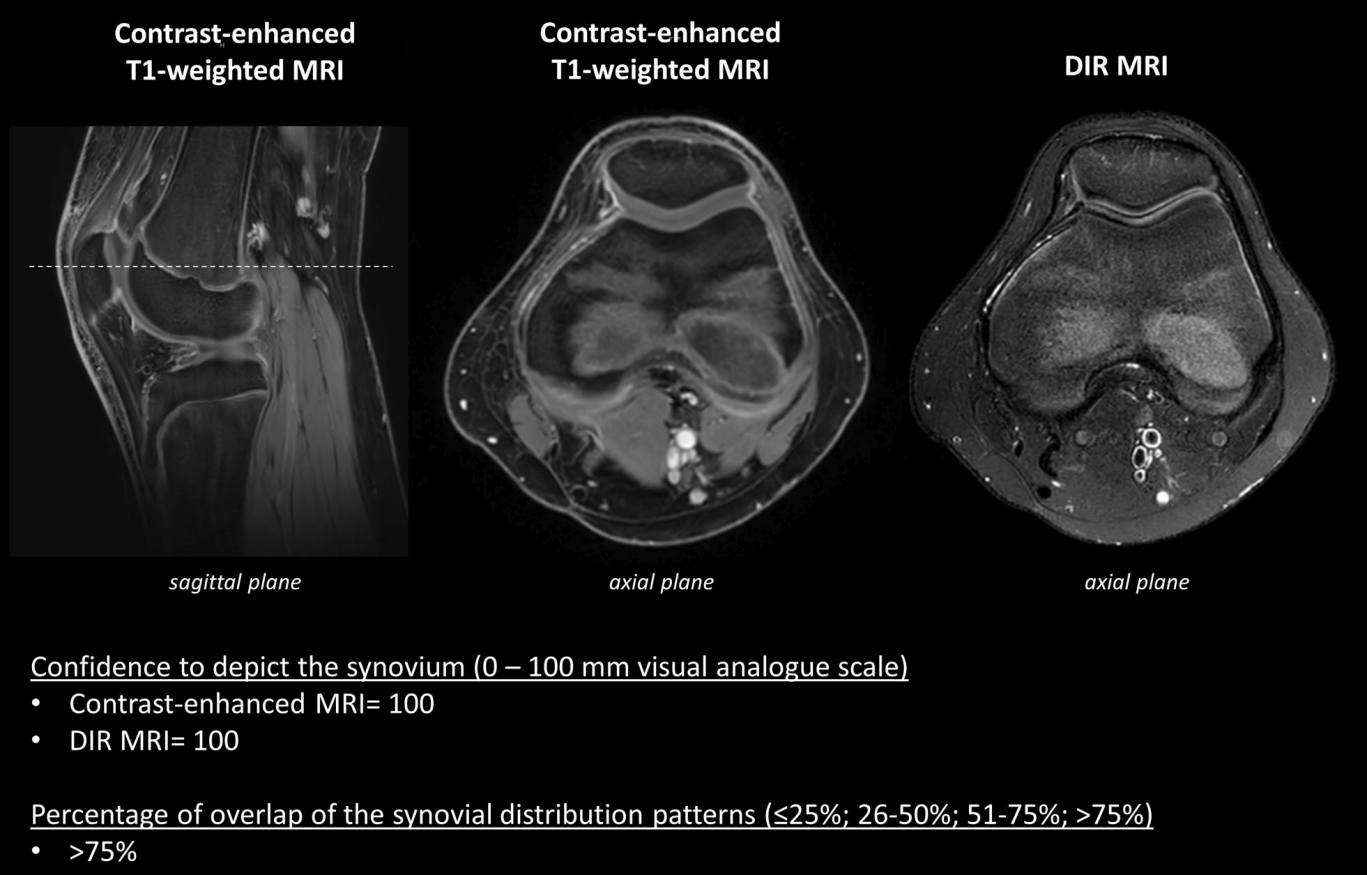
Images obtained in a 14-year-old boy, diagnosed with extended oligoarticular JIA, without knee arthritis. Confidence to depict the synovium was 100 (0–100 mm visual analogue scale) for the axial contrast-enhanced MRI and the axial DIR MRI. The percentage of overlap of synovial distribution patterns was scored as > 75%

**Fig. 3:**
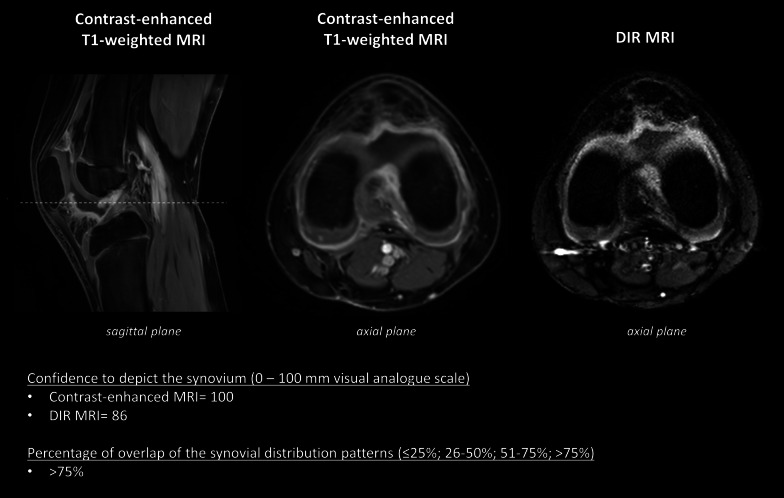
Images obtained in a 9-year-old girl, diagnosed with persistent oligoarticular JIA, with knee arthritis. Confidence to depict the synovium was 100 (0–100 mm visual analogue scale) for the axial contrast-enhanced MRI and 86 (0–100 mm visual analogue scale) for the axial DIR MRI. The percentage of overlap of synovial distribution patterns was scored as > 75%

Synovial delineation was sufficient for evaluation of all study parameters at all JAMRIS locations on DIR MRI and CE-MRI, resulting in a total analysis of 156 images per MRI technique for each reader. Examples of images of both imaging techniques obtained in children with JIA without knee arthritis and with knee arthritis are shown in Figs. 2 and 3.

### Confidence in visual identification of the synovium on DIR MRI compared to CE-MRI

Median confidence score, based on all 156 measurements, was 88.0 (IQR 79.0–97.0) for DIR MRI versus 100 (IQR 100–100) for CE-MRI (*p* value < 0.001) (Additional file 1: Table S1).

Regarding the confidence scores obtained using DIR MRI, we found no evidence of differences between the 6 JAMRIS locations (suprapatellar recesses = 92.0 [IQR 79.0–96.0]; patellofemoral = 90.0 [IQR 78.8–100]; infrapatellar fat pad = 84.5 [IQR 75.5–97.8]; cruciate ligaments = 84.0 [IQR 72.5–99.3]; medial posterior condyle = 89.5 [IQR 81.0–96.3]; lateral posterior condyle = 86.5 [IQR 79.8–96.3] [*p* value = 0.61]).

### Synovial signature on DIR MRI compared to CE-MRI

In 42/156 (26.9%) sets of paired images, the estimated percentage of overlap between the synovial distribution patterns was scored as > 75%. Examples of > 75% overlap are shown in Figs. 2 and 3. In 91/156 (58.3%) imaging sets an overlap of 51–75% was noted. An overlap of 26–50% and < 25% was observed in 15/156 (9.6%) and 8/156 (5.1%) sets of paired scans, respectively (Additional file 1: Table S2).

We found no evidence of a difference in the degree of correspondence between the 6 JAMRIS locations (location = [≤ 25% = *n* [%], 26–50% = *n* [%], 51–75% = *n* [%], 75% = *n* [%]]: suprapatellar recesses = 2 [8%], 3 [12%], 12 [46%], 9 [35%]; patellofemoral = 2 [8%], 2 [8%], 15 [58%], 7 [27%]; infrapatellar fat pad = 3 [12%], 2 [8%], 14 [54%], 7 [27%]; cruciate ligaments = 1 [4%], 4 [15%], 16 [62%], 5 [19%]; medial posterior condyle = 0 [0%], 2 [8%], 17 [65%], 7 [27%]; lateral posterior condyle = 0 [0%], 2 [8%], 17 [65%], 7 [27%] [*p* value = 0.86]).

### Maximal synovial thickness on DIR MRI compared to CE-MRI

Measurements of maximal synovial thickness on DIR MRI showed strong correlation with the findings acquired using CE-MRI (per child [aggregated measurements of 4 readers]: DIR MRI = 1.8 mm (IQR 1.3–2.5), CE-MRI = 1.8 mm (IQR 1.1–2.5), *r*_s_ = 0.93 [*p* value = < 0.001]; total measurements [aggregated measurements of 4 readers]: DIR MRI = 1.0 mm (IQR 0.0–1.6), CE-MRI = 1.0 mm (IQR 0.0–1.6), *r*_s_ = 0.91 [*p* value = < 0.001]). An overview of maximal synovial thickness measurements per reader separated by imaging technique and correlation coefficients is presented in Table 3.

**Table 3:** Inter-technique comparison: maximal synovial thickness

Reader	Maximal synovial thickness (mm)	DIR	CE-MRI	*p* *value*	*r*_s_	*p* value
Reader 1	*Per location*					
	Suprapatellar recesses	1.0 (0.0–1.4)	1.0 (0.0–1.3)	.08^a^	0.95	< .001*
	Patellofemoral	1.0 (1.0–1.6)	1.0 (1.0–1.4)	.09^a^	0.95	< .001*
	Infrapatellar fat pad	0.0 (0.0–1.3)	0.0 (0.0–1.0)	.62^a^	0.97	< .001*
	Cruciate Ligaments	1.8 (1.0–2.6)	1.6 (1.0–2.4)	.50^a^	0.89	< .001*
	Medial posterior condyle	0.0 (0.0–1.0)	0.0 (0.0–1.3)	.68^a^	0.85	< .001*
	Lateral posterior condyle	0.0 (0.0–1.0)	0.0 (0.0–1.0)	.60^a^	0.99	< .001*
	Per child	1.9 (1.0–2.8)	1.9 (1.0–2.4)	.46^a^	0.96	< .001*
	Total measurements (*n* = 156)	1.0 (0.0–1.6)	1.0 (0.0–1.4)	.44^a^	0.93	< .001*
Reader 2	*Per location*					
	Suprapatellar recesses	1.0 (0.0–1.6)	1.2 (0.0–1.7)	.67^a^	0.88	< .001*
	Patellofemoral	1.2 (1.0–1.4)	1.3 (0.8–1.7)	.18^a^	0.84	< .001*
	Infrapatellar fat pad	0.0 (0.0–1.0)	0.0 (0.0–1.2)	.82^a^	0.93	< .001*
	Cruciate Ligaments	1.5 (0.0–2.1)	1.3 (1.0–2.5)	.18^a^	0.73	< .001*
	Medial posterior condyle	0.0 (0.0–1.3)	0.0 (0.0–1.2)	.47^a^	0.85	< .001*
	Lateral posterior condyle	0.0 (0.0–1.0)	0.0 (0.0–1.0)	.27^a^	0.99	< .001*
	Per child	1.6 (1.3–2.4)	1.6 (1.0–2.6)	.53^a^	0.90	< .001*
	Total measurements (*n* = 156)	1.0 (0.0–1.4)	1.0 (0.0–1.6)	.28^a^	0.87	< .001*
Reader 3	*Per location*					
	Suprapatellar recesses	1.3 (0.0–1.6)	1.3 (0.0–1.8)	.18^a^	0.83	< .001*
	Patellofemoral	1.5 (1.3–1.8)	1.4 (0.8–1.7)	.07^a^	0.68	< .001*
	Infrapatellar fat pad	0.0 (0.0–1.7)	0.0 (0.0–1.7)	.36^a^	0.96	< .001*
	Cruciate Ligaments	1.7 (1.2–2.2)	1.8 (1.1–2.2)	.78^a^	0.84	< .001*
	Medial posterior condyle	0.0 (0.0–1.5)	0.0 (0.0–1.6)	.07^a^	0.98	< .001*
	Lateral posterior condyle	0.0 (0.0–1.0)	0.0 (0.0–1.1)	.44^a^	0.80	< .001*
	Per child	1.8 (1.5–2.5)	1.9 (1.3–2.3)	.68^a^	0.94	< .001*
	Total measurements (*n* = 156)	1.2 (0.0–1.7)	1.0 (0.0–1.8)	.36^a^	0.89	< .001*
Reader 4	*Per location*					
	Suprapatellar recesses	1.2 (0.0–1.6)	1.3 (0.0–1.7)	.16^a^	0.90	< .001*
	Patellofemoral	1.4 (1.1–2.0)	1.5 (1.0–1.9)	.64^a^	0.79	< .001*
	Infrapatellar fat pad	0.0 (0.0–1.6)	0.0 (0.0–1.5)	.69^a^	0.98	< .001*
	Cruciate Ligaments	1.5 (1.0–2.2)	1.6 (1.2–2.5)	.06^a^	0.95	< .001*
	Medial posterior condyle	0.0 (0.0–1.1)	0.0 (0.0–1.3)	.67^a^	0.99	< .001*
	Lateral posterior condyle	0.0 (0.0–1.1)	0.0 (0.0–1.2)	.11^a^	0.99	< .001*
	Per child	1.8 (1.3–2.4)	1.8 (1.3–2.6)	.86^a^	0.85	< .001*
	Total measurements (*n* = 156)	1.0 (0.0–1.8)	1.1 (0.0–1.7)	.07^a^	0.96	< .001*

Data are displayed as median (interquartile range). Data are based on 26 measurements, unless otherwise stated

*r*_s_, Spearman’s rank-order correlation; DIR, double inversion recovery; CE, contrast-enhanced

*p value < .05

^a^Wilcoxon signed-rank test

Assessment of the inter-reader reliability regarding maximal synovial thickness on DIR MRI demonstrated excellent ICC’s, which corresponded with the results obtained using CE-MRI (per child: ICC DIR MRI = 0.87 [95% CI 0.78–0.93] [*p* value = < 0.001], ICC CE-MRI = 0.90 [95% CI 0.83–0.95] [*p* value = < 0.001]; total measurements: ICC DIR MRI = 0.87 [95% CI 0.84–0.90] [*p* value = < 0.001], ICC CE-MRI = 0.87 [95% CI 0.84–0.90] [*p* value = < 0.001]) (Table 4).

**Table 4:** Inter-reader reliability: maximal synovial thickness

Maximal synovial thickness	DIR			CE-MRI		
(mm)	ICC^a^	95% CI	*p* *value*	ICC^a^	95% CI	*p* *value*
*Per location* (*n* = 104)						
Suprapatellar recesses	0.91	0.84–0.96	< .001*	0.92	0.87–0.96	< .001*
Patellofemoral	0.81	0.69–0.90	< .001*	0.85	0.76–0.92	< .001*
Infrapatellar fat pad	0.85	0.75–0.92	< .001*	0.86	0.76–0.93	< .001*
Cruciate Ligaments	0.79	0.68–0.89	< .001*	0.81	0.69–0.90	< .001*
Medial posterior condyle	0.83	0.71–0.91	< .001*	0.80	0.67–0.89	< .001*
Lateral posterior condyle	0.92	0.86–0.96	< .001*	0.92	0.87–0.96	< .001*
Per child (*n* = 104)	0.87	0.78–0.93	< .001*	0.90	0.83–0.95	< .001*
Total measurements (*n* = 624)	0.87	0.84–0.90	< .001*	0.87	0.84–0.90	< .001*

Data are based on the aggregated measurements of 4 readers

DIR, double inversion recovery; CE, contrast-enhanced; ICC, intraclass correlation coefficient; CI, confidence interval

*p value < .05

^a^Based on single-measurement, absolute-agreement, two-way random effects model

### Intra-reader reliability for maximal synovial thickness measurement on DIR MRI

Excellent ICC’s were observed for maximal synovial thickness on DIR MRI (per child: ICC reader 1 [R.H.] = 0.96 [95% CI 0.91–0.98], ICC reader 2 [K.F.v.D.] = 0.93 [95% CI 0.83–0.97], ICC reader 3 [E.E.D.] = 0.93 [95% CI 0.85–0.97], ICC reader 4 [M.M.] = 0.95 [95% CI 0.89–0.98] [*p* value [reader 1–4] = < 0.001]; total measurements: ICC reader 1 [R.H.] = 0.95 [95% CI 0.94–0.97], ICC reader 2 [K.F.v.D.] = 0.94 [95% CI 0.92–0.96], ICC reader 3 [E.E.D.] = 0.95 [95% CI 0.94–0.97], ICC reader 4 [M.M.] = 0.95 [95% CI 0.93–0.96] [*p* value [reader 1–4] = < 0.001]).

**Fig. 4:**
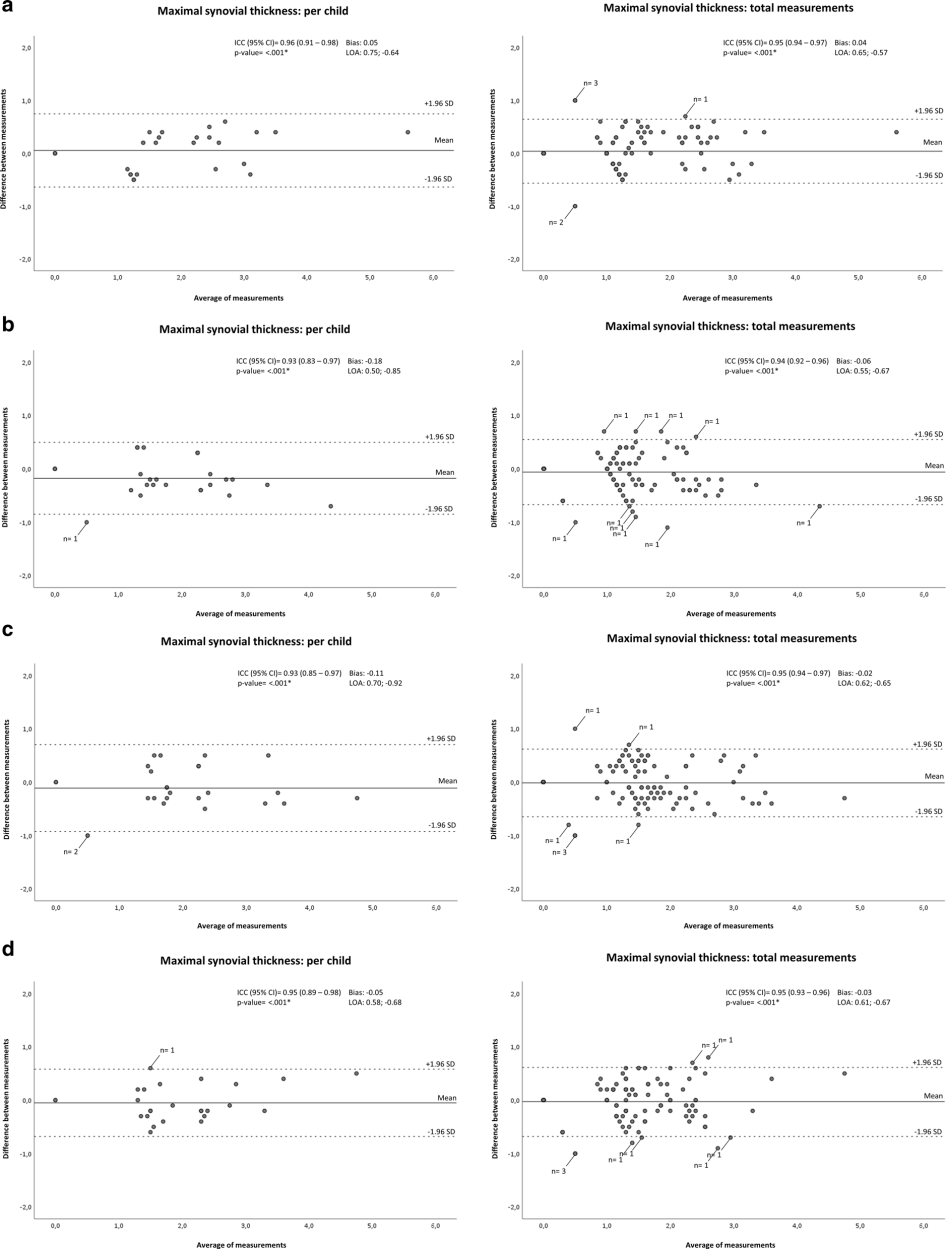
Bland–Altman plots. Bland–Altman plots (**a** = reader 1 [R.H.]; **b** = reader 2 [K.F.v.D.]; **c** = reader 3 [E.E.D.]; **d** = reader 4 [M.M.]) showing the per-child differences between the first and second measurement of maximal synovial thickness on DIR MRI. On the *x*-axis, the average of maximal synovial thickness ((measurement DIR 1 + measurement DIR 2)/2) is displayed. The *y*-axis reflects the difference between both measurements (measurement DIR 1 − measurement DIR 2). Indicated by horizontal lines are the mean of the differences (bias) and limits of agreement (LOA; + 1.96 SD and − 1.96 SD). ICC, intraclass correlation coefficient (based on single-measurement, absolute-agreement, two-way mixed effects model); CI, confidence interval; *p value < .05

Bland–Altman analyses revealed good intra-reader agreement, indicated by small mean differences (bias) and narrow limits of agreement (LOA), for all readers (per child: bias reader 1 [R.H.] = 0.05 [LOA 0.75; − 0.64], bias reader 2 [K.F.v.D.] = − 0.18 [LOA 0.50; − 0.85}, bias reader 3 [E.E.D.] = − 0.11 [LOA 0.70; − 0.92], bias reader 4 [M.M.] = − 0.05 [LOA 0.58; − 0.68]; total measurements: bias reader 1 [R.H.] = 0.04 [LOA 0.65; − 0.57], bias reader 2 [K.F.v.D.] = − 0.06 [LOA 0.55; − 0.67], bias reader 3 [E.E.D.] = − 0.02 [LOA 0.62; − 0.65], bias reader 4 [M.M.] = − 0.03 [LOA 0.61; − 0.67]) (Fig. 4).

### Maximal synovial thickness per child as measurement tool for evaluation of arthritis on DIR MRI

**Fig. 5:**
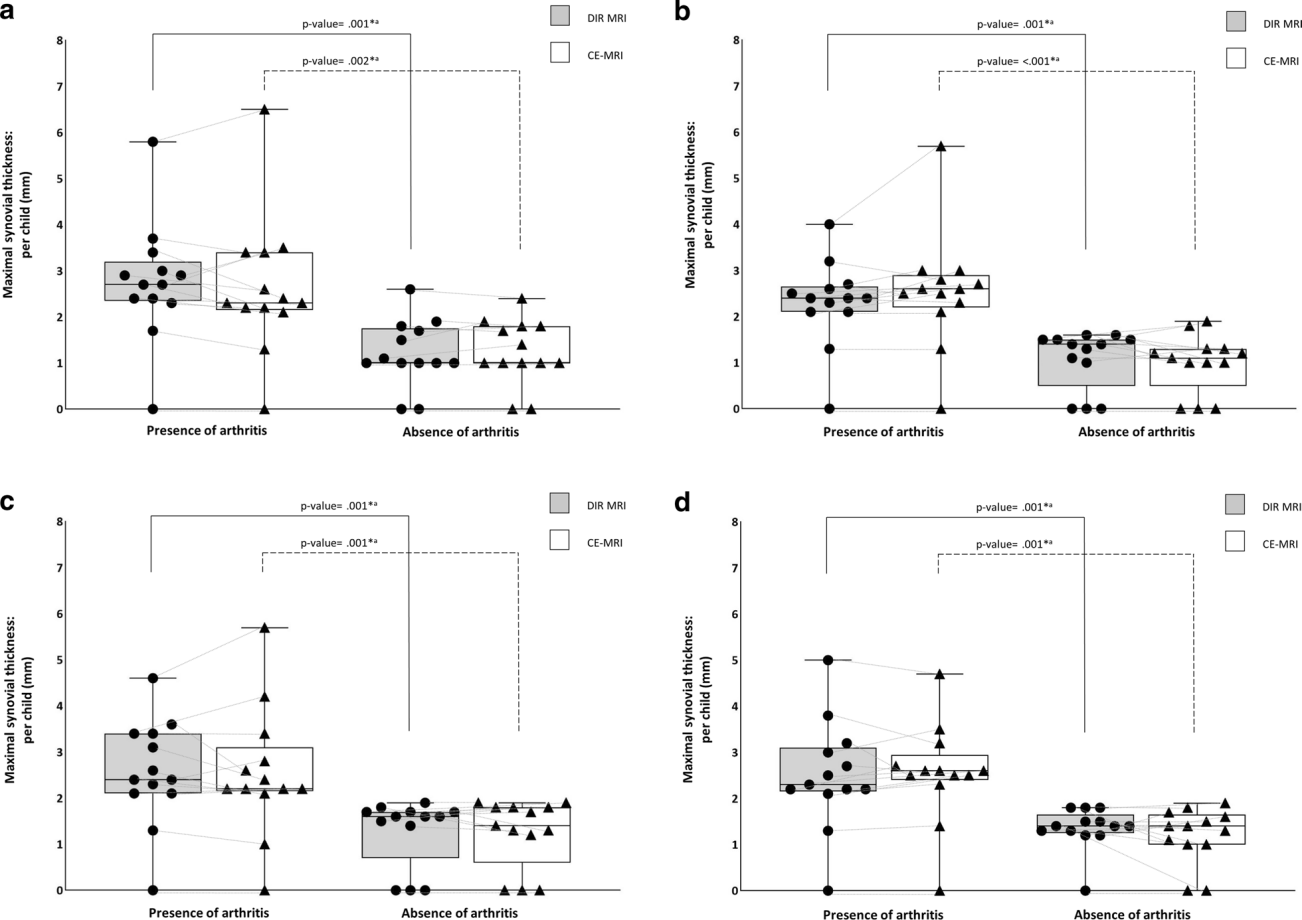
Scattered boxplots. Boxplots combined with dot density plots (**a** = reader 1 [R.H.]; **b** = reader 2 [K.F.v.D.]; **c** = reader 3 [E.E.D.]; **d** = reader 4 [M.M.]) showing the distribution of the maximal synovial thickness per child in children with JIA classified as having knee arthritis and children with JIA without knee arthritis separated by imaging technique. Whiskers indicate the maximum and minimum measured value. Lines between circles (individual measurement on DIR MRI) and triangles (individual measurement on CE-MRI) show the relationship between corresponding individual measurements. ^a^Mann–Whitney *U* test; DIR, double inversion recovery; CE, contrast-enhanced; *p value < .05

Subgroup analyses demonstrated greater maximal synovial thickness per child on DIR MRI in children affected by JIA with knee arthritis than in children with JIA without knee arthritis (per child [aggregated measurements of 4 readers]: [presence of arthritis] 2.4 mm [IQR 2.1–3.1] versus [absence of arthritis] 1.4 mm [IQR 1.0–1.6] [*p* value = < 0.001]). These results corresponded with the findings acquired using CE-MRI (per child [aggregated measurements of 4 readers]: [presence of arthritis] 2.5 mm [IQR 2.2–3.0] versus [absence of arthritis] 1.3 mm [IQR 1.0–1.8] [*p* value = < 0.001]). Figure 5a–d demonstrates boxplots for each reader showing the distribution of the maximal synovial thickness per child in children with JIA classified as having knee arthritis and children with JIA without knee arthritis separated by imaging technique.

Within the subgroups, we found no evidence of differences between the 4 readers regarding the maximal synovial thickness per child on DIR MRI ([presence of arthritis] per child: reader 1 [R.H.] = 2.7 mm [IQR 2.4–3.2], reader 2 [K.F.v.D.] = 2.4 mm [IQR 2.1–2.7], reader 3 [E.E.D] = 2.4 mm [IQR 2.1–3.4], reader 4 [M.M.] = 2.3 mm [IQR 2.2–3.1] [*p* value = < 0.18]; [absence of arthritis] per child: reader 1 [R.H.] = 1.0 mm [IQR 1.0–1.8], reader 2 [K.F.v.D.] = 1.4 mm [IQR 0.5–1.5], reader 3 [E.E.D] = 1.6 mm [IQR 0.7–1.7], reader 4 [M.M.] = 1.4 mm [IQR 1.3–1.7] [*p* value = < 0.24]).

## Discussion

Nowadays, intravenous administration of gadolinium-based contrast agents is still recommended for optimal MRI assessment of synovitis in children with (suspected) juvenile idiopathic arthritis (JIA) [7]. However, needle insertion to acquire peripheral venous access is time-consuming and experienced by children as one of the most stressful aspects of health care [8, 9]. Accordingly, there has been wide interest in non-enhanced MRI techniques that enable accurate assessment of the synovium in children. Diffusion-weighted imaging has shown its potential as child-friendly MRI technique for detection of knee arthritis in children with JIA. Although radiologists could detect synovitis on diffusion-weighted imaging with relatively high accuracy, distinction between the synovial membrane and surrounding tissues remained challenging [25]. In a recent feasibility study [21], double inversion recovery (DIR) MRI, a non-enhanced MRI technique often used for neuroimaging [11–17], demonstrated potential for delineation of the synovium. Reports on DIR MRI for assessment of the synovium are scarce and restricted to the adult knee [20–22]. In our study, we assessed the value of DIR MRI for evaluation of knee synovitis in children with JIA and compared the results to corresponding findings acquired using conventional contrast-enhanced (CE) MRI. We showed that the DIR pulse sequence adequately delineated the synovium throughout the knee in children with JIA (confidence in visual identification of the synovium was 88.0 [0–100 mm visual analogue scale] for DIR MRI; synovial signature overlap between DIR MRI and CE-MRI was 51–100% in 133/156 [85%] of the paired images). Moreover, we uncovered that measurement of maximal synovial thickness per child on DIR MRI corresponded to that of CE-MRI (*r*_s_ = 0.93 [*p* value = 0.001]; inter-reader reliability: ICC DIR MRI = 0.87 [*p* value = < 0.001], ICC CE-MRI = 0.90 [*p* value = < 0.001]) and demonstrated potential to discriminate present knee arthritis from absent knee arthritis ([presence of arthritis] 2.4 mm versus [absence of arthritis] 1.4 mm [*p* value = < 0.001]).

We evaluated the performance of DIR MRI to accentuate the synovial lining in the pediatric knee. We demonstrated that the confidence to depict the synovium was higher for conventional CE-MRI (median: 100 [IQR 100–100]) than for DIR MRI (median: 88 [IQR 79–97]). This outcome was not unexpected, since DIR MRI is, in contrast to CE-MRI, not incorporated in MRI protocols for assessment of arthritis [7, 31]. Consequently, musculoskeletal radiologists are familiar with post-contrast scans and unfamiliar with DIR-derived images of joints. Regardless, our findings indicate that DIR MRI enables fine delineation of the synovium throughout the knee. These results coincide to a great extent with the findings of the study conducted by Yi et al. [22] on a cohort of adults. In that study, good to excellent visualization of the synovium was observed in the majority of assessed images [22]. Accordingly, our study and the study by Yi et al. [22] demonstrate that musculoskeletal radiologists with different backgrounds concur in the opinion that DIR MRI enables good visualization of the synovium.

Furthermore, we observed that the synovial distribution patterns on DIR MRI and post-contrast scans are relatively similar to one another. This is in conformity with the results of the study by Son et al. [21] on a cohort of 32 adults. The finding that DIR MRI generates synovial distribution patterns that, to a great extent, overlap with the synovial signatures acquired by conventional CE-MRI is, however, not so straightforward as it might seem from anatomical perspective. To accentuate the synovial lining on DIR MRI, signals from fat tissue and effusion must be suppressed. To do so, two 180° radio-frequent pulses must be carefully applied so that the inverted longitudinal magnetization of fat and fluid reaches the null point concurrently when image acquisition occurs. Since the recovery rate of the magnetization relies on tissue-specific T1 relaxation time [18, 19], any small variation in T1 values of fat- or fluid-containing structures that surround the synovium could lead to insufficient suppression and subsequent uncertainty regarding the exact etiology of the delineated silhouette. Since the synovial distribution pattern on CE-MRI is considered the ‘optimal’ imaging reflection of ‘true’ synovial distribution [7, 31], the good inter-technique agreement indicates that the relatively increased signal intensity on DIR MRI represents solely the synovium. Nevertheless, in a small percentage of the DIR/CE-MRI image pairs (i.e., 5.1%) we observed < 25% overlap of synovial distribution patterns. This finding might be the result of magnification of the margins of the enhanced signature on some of the post-contrast scans due to gadolinium leakage from the synovium into the adjacent joint fluid prior to image acquisition [32]. Accordingly, the hypothesis arises that the synovial figure derived from DIR MRI might be a more accurate correlate of ‘actual’ synovial distribution.

In this study, we also demonstrated that synovial thickness measurements on DIR MRI strongly correlated with CE-MRI measures. Son and co-workers also reported strong correlation coefficients, but, in contrast to our results, they measured significantly thicker synovium on DIR MRI than on CE-MRI [21]. The authors hypothesized that this might be the result of some structural overestimation of the distance between the synovial borders secondary to the relatively low spatial resolution on the derived DIR MRI [21]. We might have overcome this issue by applying a different technique for scanning time reduction. In the study by Son et al. [21], exclusively sensitivity encoding (SENSE) was used, whereas we used a combination of SENSE and compressed sensing (Compressed SENSE) to obtain a relatively higher spatial resolution [33]. Consequently, synovial borders might have been identified more easily in our study. Evaluation of synovial thickness on conventional CE-MRI, in accordance with the JAMRIS system, is currently the only validated semi-quantitative method for assessment of JIA disease activity in the knee [26]. In our study, significantly greater DIR-derived maximal synovial thickness values were observed in children with JIA with knee arthritis compared to children with JIA without knee arthritis. These results corresponded with the findings acquired by CE-MRI, suggesting similar performance of DIR MRI and CE-MRI in assessment of pathologic synovial thickening. This is in line with the findings of Yi et al. [22], as the authors demonstrated no significant difference between synthetic DIR MRI and conventional CE-MRI regarding the detection of synovitis (i.e., synovial thickness > 2 mm).

Replacement of CE-MRI by contrast-free DIR MRI is expected to break down the current barriers to MRI examination of the synovium in the pediatric population. Accordingly, this will considerably expand the horizons of MRI as diagnostic tool as well as marker of disease activity in children with rheumatologic disorders. Moreover, DIR MRI might be the key to accomplish the long-cherished goal of developing reference values for the synovial thickness for every single JIA-relevant joint [34, 35], as it could pave the way for future studies to assess the physiological appearance of the synovium in multiple joints in large cohorts of completely healthy children of all age-stages.

This study had some limitations. First, we included a relatively small number of children. Second, since the median age of the included children was 14 years (IQR 11–17), the study findings might not be fully applicable to the youngest category of children with JIA. Third, post-contrast image acquisition was not completely standardized (i.e., CE-MRI scans were obtained < 5 min after contrast injection). Therefore, the results regarding the overlap of synovial distribution patterns might be affected to some extent by small time-dependent enhancement variability [29, 30]. Fourth, DIR MRI was acquired in the axial plane only. Accordingly, study parameters were not assessed from the perspective of different slice directions. Consequently, reader’s orientation, with respect to the synovial lining on DIR scans, might be restricted to some extent. Finally, identification of synovial thickening without active inflammation might be challenging on DIR MRI, as the DIR pulse sequence is not expected to generate sufficient difference in signal intensity between inflamed (hypervascular) synovium and inactive (hypovascular) fibrous ‘pannus.’ However, as it is recommended to avoid interpretation of synovial signal intensity on static CE-MRI (the current MRI technique of choice [1–4, 7]) for evaluation of JIA disease activity in the knee [36], the potential advantage of static CE-MRI regarding the above-mentioned issue is expected to be minimal. Since diffusion of water molecules is expected to be restricted within inactive fibrous ‘pannus’ as opposed to inflamed well-vascularized synovial tissue, a multitechnique analysis of the synovium by using synovial thickness measurement on DIR MRI and diffusion-weighted imaging with apparent diffusion coefficient mapping could unlock the full potential of contrast-free MRI for evaluation of synovitis in children with pediatric rheumatologic disorders. Nevertheless, future studies are necessary to corroborate this hypothesis.

## Conclusions

DIR MRI adequately delineated the synovial lining and enabled adequate evaluation of the synovium in the knee in children with JIA. Synovial thickness measurement using DIR MRI provided values that strongly correlated with conventional CE-MRI measures and exhibited potential to discriminate present knee arthritis from absent knee arthritis. Our results suggest that DIR MRI should be considered as the child-friendly alternative to CE-MRI for evaluation of synovitis in children with (suspected) JIA. To explore the full potential of DIR MRI for assessment of synovitis, future studies should include children of all age-stages and consider acquisition of images in multiple slice directions.

## Supplementary Information

**Additional file 1: Supplemental Table 1.** Confidence in visual identification of the synovial membrane on DIR MRI compared to CE-MRI. **Supplemental Table 2.** Correspondence between DIR MRI and CE-MRI regarding the synovial distribution patterns.

## Data Availability

The datasets generated and/or analyzed during the current study are not publicly available, but are available from the corresponding author on reasonable request.

## Abbreviations

CE: Contrast enhanced
DIR: Double inversion recovery
ICC: Intraclass correlation coefficient
JAMRIS: Juvenile arthritis MRI scoring
JIA: Juvenile idiopathic arthritis
