# Supplementary material for: Double inversion recovery MRI versus contrast-enhanced MRI for evaluation of knee synovitis in juvenile idiopathic arthritis
Source: Insights Imaging. 2022 Oct 20;13:167. doi: 10.1186/s13244-022-01299-0 (PMC9584003; doi:10.1186/s13244-022-01299-0)
Supplement: Supplementary file 1 — Additional file 1: Supplemental Table 1. Confidence in visual identification of the synovial membrane on DIR MRI compared to CE-MRI. Supplemental Table 2. Correspondence between DIR MRI and CE-MRI regarding the synovial distribution patterns. [file 13244_2022_1299_MOESM1_ESM.docx]

**Supplemental Table 1.** *Confidence in visual identification of the synovial membrane on DIR MRI compared to CE-MRI*

| Confidence in visual |  | |  |  |
| --- | --- | --- | --- | --- |
| identification of the synovium | DIR | CE-MRI | *p-value* |  |
| *Per location*  Suprapatellar recesses  Patellofemoral  Infrapatellar fat pad  Cruciate Ligaments  Medial posterior condyle  Lateral posterior condyle  Total measurements (n= 156) | 92.0 (79.0 – 96.0)  90.0 (78.8 – 100)  84.5 (75.5 – 97.8)  84.0 (72.5 – 99.3)  89.5 (81.0 – 96.3)  86.5 (79.8 – 96.3)  88.0 (79.0 – 97.0) | 100 (100 – 100)  100 (100 – 100)  100 (100 – 100)  100 (100 – 100)  100 (100 – 100)  100 (100 – 100)  100 (100 – 100) | .001*^a^  .001*^a^  <.001*^a^  <.001*^a^  <.001*^a^  <.001*^a^  <.001*^a^ |  |
|  |  |  |  |  |

Data is displayed as median (interquartile range). Data is based on 26 measurements, unless otherwise stated. ^a^ Wilcoxon signed-rank test; Confidence was measured on a visual analogue scale: 0 mm= no confidence, 100 mm= maximal confidence; DIR= Double Inversion Recovery; CE= Contrast-Enhanced; * p-value <.05.

**Supplemental Table 2.** *Correspondence between DIR MRI and CE-MRI regarding the synovial distribution patterns*

| Synovial signature on DIR MRI | Percentage of overlap | | | |  |
| --- | --- | --- | --- | --- | --- |
| compared to CE-MRI  *Per location*  Suprapatellar recesses  Patellofemoral  Infrapatellar fat pad  Cruciate Ligaments  Medial posterior condyle  Lateral posterior condyle | ≤25%  2 (8%)  2 (8%)  3 (12%)  1 (4%)  0 (0%)  0 (0%) | 26-50%  3 (12%)  2 (8%)  2 (8%)  4 (15%)  2 (8%)  2 (8%) | 51-75%  12 (46%)  15 (58%)  14 (54%)  16 (62%)  17 (65%)  17 (65%) | >75%  9 (35%)  7 (27%)  7 (27%)  5 (19%)  7 (27%)  7 (27%) |  |
| Total measurements (n= 156) | 8 (5%) | 15 (10%) | 91 (58%) | 42 (27%) |  |

Data is displayed as frequency (percentage). Data is based on 26 measurements, unless otherwise stated.
